# Modeling the repetitions‐in‐reserve‐velocity relationship: a valid method for resistance training monitoring and prescription, and fatigue management

**DOI:** 10.14814/phy2.15955

**Published:** 2024-02-28

**Authors:** Ivan Jukic, Katarina Prnjak, Eric R. Helms, Michael R. McGuigan

**Affiliations:** ^1^ Sport Performance Research Institute New Zealand (SPRINZ) Auckland University of Technology Auckland New Zealand; ^2^ School of Engineering, Computer and Mathematical Sciences Auckland University of Technology Auckland New Zealand; ^3^ Division of Sport and Exercise Sciences, School of Applied Sciences Abertay University Dundee United Kingdom; ^4^ School of Medicine Western Sydney University Sydney Australia; ^5^ Graduate School of Health University of Technology Sydney Sydney Australia

**Keywords:** exercise monitoring, exercise prescription, fatigue, rating of perceived exertion, strength training, velocity‐based training

## Abstract

Establishing a relationship between repetitions left in reserve and the mean absolute velocity (RIR‐velocity relationship) during resistance training (RT) could allow for objective monitoring, prescription, and real‐time adjustment of the training load and set‐volume. Therefore, we examined the goodness of fit and prediction accuracy of general and individual RIR‐velocity relationships in the free‐weight back squat exercise. The effects of sex, training status and history, as well as personality traits, on the goodness of fit and the accuracy of these relationships were also investigated. Forty‐six resistance‐trained people (15 females and 31 males) performed a one‐repetition maximum (1RM) test, and two repetitions to failure (RTF) tests 72 h apart. We found greater goodness of fit of individual RIR‐velocity relationships compared to general RIR‐velocity relationships. Individual, but not general RIR‐velocity relationships established in the first testing session yielded acceptable prediction accuracy of RIR (mean error <2 repetitions) in the subsequent testing session, regardless of the load used. Similar results were obtained when both general and individual RIR‐velocity relationships were averaged across the loads, suggesting that a single RIR‐velocity relationship covering a range of loads can be used instead of traditional RT methods, potentially allowing for better fatigue management and more efficient adaptation.

## 1 INTRODUCTION

Resistance training (RT) is often performed by athletes to improve muscle strength, power, and hypertrophy as these adaptations often coincide with improvements in jumping, sprinting, change of direction, and sport‐specific performance (Suchomel et al., 2016, 2018). In addition, RT is also recommended to the general population due to its profound, positive effects on physical and mental performance, health, and quality of life (Feigenbaum & Pollock, 1999; Kraemer et al., 2002; O'Connor et al., 2010). Many variables come into play when designing an efficacious RT program such as exercise selection, order, frequency, intensity, volume, velocity, and set structure, among others. However, training intensity and volume receive comparably more attention from the scientific literature as the manipulation of these training variables often determines the magnitude of RT‐induced adaptations.

RT intensity is historically prescribed by selecting the load relative to the individual's one‐repetition maximum (1RM). On the contrary, RT volume is often defined as the number of sets and repetitions performed in a training session. Thus, when the number of sets for a training session is fixed, volume is prescribed and largely determined by the number of repetitions performed per set (i.e., set‐volume). Set‐volume prescription is often based on the theoretical relationship between the maximum number of repetitions individuals can do with a given percentage of 1RM (%1RM); thus, presenting an inherent connection between traditional RT intensity and volume prescription. While this RT prescription approach is relatively simple and ideal for implementation in practice, there are several limitations to it. Firstly, the 1RM test can be physically and psychologically demanding and time consuming and can also compromise the safety of an individual doing the test (Niewiadomski et al., 2008). Additionally, while 1RM tests are generally reliable (Grgic et al., 2020), changes in other factors that are challenging to control such as mood state (Rathschlag & Memmert, 2013) and multimedia exposure immediately prior to (Cook & Crewther, 2012) and poor sleep (Craven et al., 2022) the day prior to testing can impact strength performance. Thus, if a 1RM test happens to be reflective of abnormal performance, positive or negative, subsequently prescribed RT intensity would be lighter or heavier than needed to achieve desired training adaptations. Likewise, even if a 1RM test does accurately reflect the current strength levels of an individual, using this test to continuously prescribe RT intensity can be erroneous as it does not account for inherent variation in human performance due to normal biological and psychological variability and factors such as sleep (Bulbulian et al., 1996), nutrition (Helms et al., 2015), and life stress (Bartholomew et al., 2008), all of which can affect RT performance. Secondly, the maximal number of repetitions that can be completed to failure with different exercises and loads varies substantially among individuals (Richens & Cleather, 2014). This suggests that prescribing the same relative load with a fixed number of repetitions will likely lead to heterogeneous training stimuli across individuals. To combat these fundamental issues with traditional RT intensity and volume prescription, sport scientists have proposed a velocity‐based approach to monitoring and prescribing RT programs (Weakley et al., 2020).

The rationale behind a velocity‐based approach to RT intensity prescription relies on the strong, inverse relationships between barbell velocity and the %1RM observed in many multi‐joint exercises when individuals perform repetitions with maximal intent (Weakley et al., 2020). Indeed, this relationship is reliable in both Smith machine and free‐weight exercises (Banyard et al., 2018; García‐Ramos et al., 2019; Pestaña‐Melero et al., 2018), and thus useful for prescribing RT intensity by adjusting the absolute load to match the velocity associated with the intended %1RM intended for a given training session. For set‐volume prescription, a velocity‐based approach may also be beneficial as the barbell velocity decreases within a set when the exercise is performed with maximal lifting intent and fatigue ensues (Sánchez‐Medina & González‐Badillo, 2011). Indeed, a very close relationship between velocity loss (VL) and the percentage of performed repetitions out of the maximum possible in a set was observed for different exercises performed (González‐Badillo et al., 2017; Sánchez‐Moreno et al., 2017). According to these studies, when a 30% VL in a set is reached, the individuals have completed approximately 50% and 60% of the maximum number of repetitions in the Smith machine bench press and free‐weight pull‐up exercise, respectively (González‐Badillo et al., 2017; Sánchez‐Moreno et al., 2017). Unlike the load–velocity relationship, the relationship between VL and the percentage of completed repetitions suffers from several methodological issues (García‐Ramos et al., 2021; Jukic et al., 2022). For instance, the exact number of repetitions performed, or left in reserve after completing a set remains unknown, and the percentage of the completed repetitions for a given VL could vary across individuals, exercises, and loads (González‐Badillo et al., 2017; Rodríguez‐Rosell et al., 2020). Thus, current velocity‐based approaches offer advantages for RT intensity prescription but may be of limited use for RT volume prescription.

One of the most simplistic ways to terminate sets based on fatigue accumulation during RT is using the repetitions in reserve‐based rating of perceived exertion (RIR‐based RPE) scale (Zourdos et al., 2016). More specifically, individuals could be instructed to terminate sets based on the number of additional repetitions they believe they could complete before reaching muscle failure. Thus, when the load and number of sets are fixed for a given session, individuals can perform repetitions until reaching a predetermined RIR intended for inducing the desired training stimulus. Although this relatively novel method of autoregulation during RT is very practical and useful, it may be problematic since it is subjective, such that RIR accuracy is highest among those with sufficient RT experience when training closer to failure with moderate‐to‐low repetition sets (Ormsbee et al., 2019; Zourdos et al., 2021). To address this problem, modeling the RIR‐velocity relationship could be useful as it combines both velocity‐based and RIR‐based approaches to RT monitoring and prescription while relying only on the well‐established VL increase as the number of repetitions in a set increase. The establishment of RIR‐velocity relationships may enable more precise daily load prescriptions and provide real‐time insight into individuals' proximity to failure during training sessions. This enhanced understanding could empower coaches and athletes to exert greater control over the physiological stimuli applied during RT, potentially fostering more targeted and effective training adaptations.

Only two studies (García‐Ramos et al., 2018; Morán‐Navarro et al., 2019) attempted to establish the relationship between RIR and velocity. Morán‐Navarro et al. (2019) examined the within‐individual variability for the velocity associated with a given number of RIR in the Smith machine bench press, shoulder press, bench pull, and back squat. Although the authors concluded that velocity at a given RIR is very similar and highly reliable for a given exercise, noteworthy within‐individual variability was observed, especially for the bench press and shoulder press exercises and less experienced individuals. Similarly, García‐Ramos et al. (2018) also reported large between‐individual variability for the velocity at a given RIR (from 1 to 10). Based on these findings, it seems that a RIR‐velocity relationship, like a load–velocity relationship, is highly individual, though this is yet to be confirmed. However, it is important to note that RIR‐velocity relationships have not been established for free‐weight exercises, despite their popularity among trainees, especially athletes. Additionally, little is known about how sex, modeling strategies (e.g., linear vs. polynomial regression models), training history and strength levels affect the stability of RIR‐velocity relationships. Personality traits such as emotional stability and conscientiousness could also potentially affect the stability of RIR‐velocity relationships as these traits generally affect how people cope with fatigue (De Vries & Van Heck, 2002). Neuroticism—a trait on the opposite spectrum of emotional stability—represents the propensity to experience negative emotions and distress (Widiger, 2017), and as such may lead to a decreased tolerance for fatigue during exercise. Conversely, conscientiousness represents the propensity to be persistent and self‐controlled (Widiger, 2017), and as such may enhance an individual's ability to tolerate fatigue during exercise. This could be particularly relevant during RT whereby repetitions are performed with maximal intent and to (or near) muscle failure. Hypothetically, individuals who score higher on neuroticism (or lower on emotional stability) could have greater variability in movement velocity and associated RIR during a heavy RT set due to their reduced ability to cope with fatigue. Similarly, performing the concentric phase of the lift with maximal intent might be another hurdle for those individuals. In contrast, highly conscientious individuals could leverage their persistence to complete the task while giving their absolute best, ensuring the stability of their RIR‐velocity profiles. Importantly, the predictive validity of RIR‐velocity relationships—as well as factors affecting it—should also be examined to determine the usefulness of this method for RT monitoring and prescription in practice. Therefore, it is evident that many factors related to the RIR‐velocity relationship are yet to be explored.

To consider the abovementioned scarcity in the literature, this study aimed to (1) examine the goodness of fit of general and individual RIR‐velocity relationships fitted with linear and polynomial regression models in a free‐weight back squat exercise; (2) determine the effects of sex, training status and history, as well as personality traits on the models' fit; (3) determine the ability of general and individual RIR‐velocity relationships to predict data in a subsequent testing session and examine the factors potentially affecting the accuracy of the predictions; and (4) determine whether the RIR‐velocity relationship provides acceptable prediction accuracy by averaging velocities of repetitions associated with RIRs that participants achieved with more than a single load. Based on the high interindividual variability of velocity at different RIR reported in the previous two studies (García‐Ramos et al., 2018; Morán‐Navarro et al., 2019), we hypothesized greater goodness of fit for individual RIR‐velocity relationships, whereas no other hypotheses were formulated due to the dearth of literature on the topic.

## 2 METHODS

### 2.1 Design

This study is part of a larger project investigating the validity of different velocity‐based RT monitoring and prescription methodologies. Participants reported to the laboratory on four occasions with 48–72 h of rest between the sessions. In the first session, after participants' anthropometric measures were taken, participants were familiarized with the free‐weight back squat movement, the equipment, instruments, verbal instructions to move the barbell up as fast as they can, and visual barbell velocity feedback on the TV screen. In the second session, participants performed an incremental loading (i.e., 1RM) test in the back squat exercise. In the final two sessions, participants completed repetitions to failure (RTF) tests in the same exercise with 90%, 80%, and 70% of their established 1RM. All sessions were performed at the same time of the day for each participant (± 1 h), with the same researchers present, and under similar environmental conditions (∼20°C and ∼60% humidity) to control for the potential effects of these factors on performance.

### 2.2 Participants

Fifty‐one strength‐trained people (15 females and 36 males; 18–40 years of age) participated in this study. Three male participants withdrew from the study due to injuries during their work or recreational sporting activity not related to the study, whereas two male participants dropped out of the study due to undisclosed personal reasons after completing one and three experimental sessions. Since the primary aim of this study was to determine the validity of general and individual RIR‐velocity relationships, only data from participants who completed all sessions (*N* = 46) were retained for the analysis. The 1RM in the free‐weight back squat exercise was 83.29 ± 19.91 kg (relative strength = 1.25 ± 0.30) and 149.30 ± 23.66 kg (relative strength = 1.79 ± 0.35) for females and males, respectively. To be eligible for this study, participants had to confirm they (1) were willing to abstain from any additional lower‐body training during the study; (2) were not currently taking medication that would alter metabolic or cardiovascular functions; (3) had no musculoskeletal limitations; (4) were not currently using anabolic steroids or had a history of use; and (5) had at least 6 months of RT experience training at least 2×/week including the back squat exercise at least 1×/week, with no longer than 2 weeks in a row off training during that period. Each participant provided written informed consent prior to commencing the study. The protocol of this study was approved by and was in accordance with the ethical requirements of the Auckland University of Technology Ethics Committee (approval number: 20/55).

### 2.3 Familiarization session

Participants first completed a questionnaire related to their training history and usual RT practices upon arriving to the laboratory (Appendix S1). They were also asked to provide logs of their most recent (and heaviest) back squat sessions and to conservatively estimate their 1RM. This information was later used to prescribe the loads for familiarization with the lifting instructions and for warm‐ups during the upcoming 1RM session. Next, participants' body mass and height were measured using an electronic column scale and a wall mounted stadiometer (Seca Ltd., Hamburg, Germany). Participants then completed a standardized warm‐up consisting of cycling at 100 rpm for 5 min, dynamic stretching for 2 min, 10 bodyweight lunges and squats, as well as 10 barbell‐only squats. Thereafter, participants were familiarized with the instruction to lift the barbell up as fast as they can (during the concentric muscle action), feedback on the screen indicating velocity of the barbell, and the instruction to have at least a momentary pause at the top of the movement not lasting longer than 2 s between repetitions with feet maintaining contact with the floor (i.e., no jumping or lifting of the heels) at all times. To ensure familiarity with these instructions and general conditions for the 1RM and RTF tests, participants then completed three repetitions at 20%, 40%, and 60% of their estimated 1RM, and then 10 repetitions at 60% of their estimated 1RM with at least 3 min of rest between the sets. During these repetitions, participants were provided the abovementioned instructions as well as visual feedback indicating the velocity of the barbell. At the end of each session, all participants understood and felt comfortable with these conditions, and performed at least two sets with consistent repetition velocities (± 0.02 m/s). Finally, participants were instructed to keep their habitual hydration, nutrition, and caffeine practices before every subsequent testing session the same.

### 2.4 One repetition maximum session: Day 2

During the 1RM session, participants completed the same standardized warm‐up as in the familiarization session. The 1RM assessment was performed using a 20‐kg barbell (Rogue, Columbus, Ohio, USA) and calibrated weight plates (Eleiko; Halmstad, Sweden, EU). The 1RM protocol consisted of three repetitions each at 20%, 40%, and 60%, and one repetition at 80%, and 90% of an estimated 1RM, followed by 1RM attempts (Banyard et al., 2018, 2020; Jukic et al., 2020a, 2020b). After each successful attempt, the load was increased in consultation with the participant from 1 to 12.5 kg until no further weight could be lifted or until movement technique was compromised. In addition, a maximum of five 1RM attempts were allowed for each participant. Three and four minutes of passive rest were provided between each submaximal set and 1RM attempt, respectively. Participants always adopted a self‐selected foot stance and eccentric tempo. Strong verbal encouragement and visual feedback were provided throughout all trials. Participants were required to reach a squat depth at which the tops of the thighs were at least parallel to the floor, as determined by the investigators and a camera positioned perpendicularly to the participant, for a repetition to be considered successful.

### 2.5 Repetitions to failure sessions: Days 3 and 4

The same standardized warm‐up as in the familiarization and 1RM sessions was performed during the RTF sessions. Participants then completed four sets of 10, 5, 3, and 1 repetition of the free‐weight back squat exercise against 30%, 50%, 70%, and 90% of the 90% of their established 1RM (i.e., the heaviest load to be lifted that day), respectively. They were provided with 3 min of rest between warm‐up sets, and 4 min between the last warm‐up set and the first set to failure. After the general and specific warm‐up, participants performed three sets with 90%, 80%, and 70% 1RM, respectively, to failure with 10 min of rest (García‐Ramos et al., 2018; Miras‐Moreno et al., 2022; Pérez‐Castilla et al., 2023) between sets. Since the excessive fatigue from performing a high number of repetitions during RTF with 70% 1RM could have compromised the number of repetitions performed during subsequent RTF sets with 80% and 90% of 1RM, the loads were not tested in a randomized fashion. Instead, participants always performed RTF with the highest load (i.e., 90% 1RM) first while the last RTF set was always performed with the lowest load (i.e., 70% 1RM). With regards to the exercise execution (including lifting instructions, encouragement, and visual feedback), the same conditions applied as during the 1RM session. The rest between two successive RTF sessions was 72 h.

### 2.6 Data acquisition

The training history and RT practices questionnaire can be found in the Appendix S1. Briefly, the questions were related to the experience participants have with RT, in years, and the average (1) number of repetitions they perform during their own training; (2) intensity of load at which they train; and (3) number of RIR they have left after completing their training sets. As the questions were multiple‐choice; participants' responses were treated as categorical. After inspecting frequencies of responses for each category (both overall and within levels of outcome variables used in models), these variables were recorded by merging some response options to avoid having less than five responses in more than 20% of cells/categories (Field et al., 2012). In particular, the number of repetitions performed was transformed into a categorical variable with three levels (1–5, 5–8, and >8), as well as was the intensity of load (<70, 70–80, and 80–90), whereas the number of RIR and the experience with RT were transformed into categorical variables with two levels (0–2 and 2–4; ≤3 and >3, respectively).

In this study, mean velocity of all repetitions was monitored using the GymAware (GymAware, Kinetic Performance Technologies, Canberra, Australia) linear position transducer (LPT). An LPT was placed on both sides of the barbell perpendicular to the position between the hands and the loaded barbell sleeves, according to the manufacturer's instructions. The reliability and validity of this LPT have been previously confirmed and its characteristics described elsewhere (Banyard et al., 2017; Orange et al., 2020). Data obtained from the LPT were transmitted via Bluetooth to a tablet (iPad, Apple Inc., California, USA) using the GymAware v2.8.0 app. LPT attached to the right side of the barbell was connected to the TV and provided visual feedback indicating velocity of the barbell after each repetition. The data from this LPT was used for the analysis. Finally, to avoid any data loss due to issues with online clouds or the internet connection, mean velocity of all repetitions were manually recorded and organized in a Microsoft Excel spreadsheet (Microsoft Corporation, Redmond, Washington, USA) during each session. To ensure consistency and accuracy of this procedure, the same two researchers handled this task throughout the study.

International Personality Item Pool (IPIP) Big Five Personality Inventory was used to assess stable personality traits, namely, Agreeableness, Conscientiousness, Extraversion, Neuroticism, and Openness (Ehrhart et al., 2008; Goldberg, 1992). The inventory contains 50 statements (items), 10 for each of the mentioned Big Five personality dimensions which were administered using the Qualtrics survey‐building software. Participants had to estimate how well each statement describes them using a 5‐point Likert‐type scale ranging from 1 (*very inaccurate*) to 5 (*very accurate*). Scores were averaged across items to obtain total scores for each of the five personality dimensions. For the purposes of the present study, only conscientiousness and emotional stability were used in the analysis.

### 2.7 Statistical analysis

Linear and second order polynomial regression models were used to fit the general and individual RIR‐velocity relationships. General RIR‐velocity relationships were established separately for each load and testing session by pooling together the data from all participants, whereas individual relationships were determined for each participant separately, on both testing sessions and with each load. The goodness of fit of general relationships was examined through the coefficient of determination (*R* ^*2*^) and residual standard error (*RSE*) of the models, whereas medians and ranges of *R* ^*2*^ and *RSE* were evaluated for individual RIR‐velocity relationships.

Linear mixed‐effects models with the Gaussian conditional distribution and identity link function were used to examine factors influencing the goodness of fit of individual RIR‐velocity relationships. For this purpose, the load (3 levels), training experience (2 levels) and practices (2 or 3 levels), relative strength and conscientiousness and emotional stability were all considered as fixed effects.

The predictive validity of the general and individual VL‐%_repetitions_ relationships was examined by using the models from the first testing session (i.e., a general model with all participants' data pooled and individual models of each participant) and fitting them to the data of the second testing session. Thereafter, general, and individual models' errors were evaluated by calculating an absolute difference between the observed and predicted data in the second testing session. Models' absolute error of less than one repetition was deemed excellent, 1–2 repetitions acceptable, and more than two repetitions error not useful for monitoring and prescribing RT. This decision was made given the existence of other well‐established, cost‐free methods of monitoring and prescribing RT, such as stopping sets at a predetermined perceived number of RIR (Halperin et al., 2021). To examine factors which influenced the absolute differences between observed and predicted data, linear mixed‐effects models were used, as previously described. Finally, to confirm the robustness of these findings, generalized linear mixed‐effects models, with a binomial conditional distribution and logit link function, were also used to examine factors affecting the probability of not exceeding an absolute prediction error of two repetitions.

For all mixed models, participants (*n* = 46) were treated as random effects to control for repeated measurements, and the general variation between participants. Since both fixed and random effects were used, restricted maximum likelihood estimation was used for evaluation of the linear mixed‐effects models whereas maximum likelihood, with Laplace approximation, estimation was used for generalized linear mixed‐effects models. The contribution of both fixed and random effects to the explanatory power of any of the explored models was examined using a likelihood ratio test, deviance statistic, and Akaike Information Criterion score, before selecting the final model to obtain the best fit while maintaining model parsimony. Importantly, the reduction of the model structure was always theoretically motivated and was done as a last resort. The statistical significance of fixed effects was examined by *t*‐tests based on the Satterthwaite approximation or Wald *Z*‐tests for linear mixed‐effects and generalized linear mixed‐effects models, respectively. For linear mixed‐effects models, predictors' estimates and 95% confidence intervals (95% CI) were calculated and presented whereas for generalized mixed‐effects models odds ratios with associated 95% CI were evaluated and presented to aid the interpretation of the findings. For categorical predictors with more than two levels, post hoc tests were performed with Holm‐Bonferroni correction. More details on models' specification and diagnostics can be found in Appendix S2.

To explore whether RIR‐velocity relationships could be further simplified, both general and individual RIR‐velocity relationships were also established by averaging velocities of repetitions associated with RIRs that participants achieved with more than a single load. For instance, if an individual performed 5, 10, and 15 repetitions with 90%, 80%, and 70% of 1RM, respectively, velocities of RIRs from 1 to 5 and 6 to 10 were averaged across three and two loads, respectively. Thereafter, their goodness of fit and prediction validity was assessed as previously described for RIR‐velocity relationships for each load separately.

All statistical analyses were performed in R language and environment for statistical computing (version 4.2.0, The R foundation for Statistical Computing, Vienna, Austria) using the *lme4* (Bates et al., 2015) and *ggeffects* (Lüdecke, 2018a) packages, models' performance using the *performance* and *DHARMa* (Hartig, 2020) packages, and preparation and visualization of data using the *tidyverse* (Wickham et al., 2019) and *sjPlot* (Lüdecke, 2018b) packages. Custom‐written R script and associated dataset are available at the Open Science Framework repository (URL: https://osf.io/5ejcp/).

## 3 RESULTS

**FIGURE 1:**
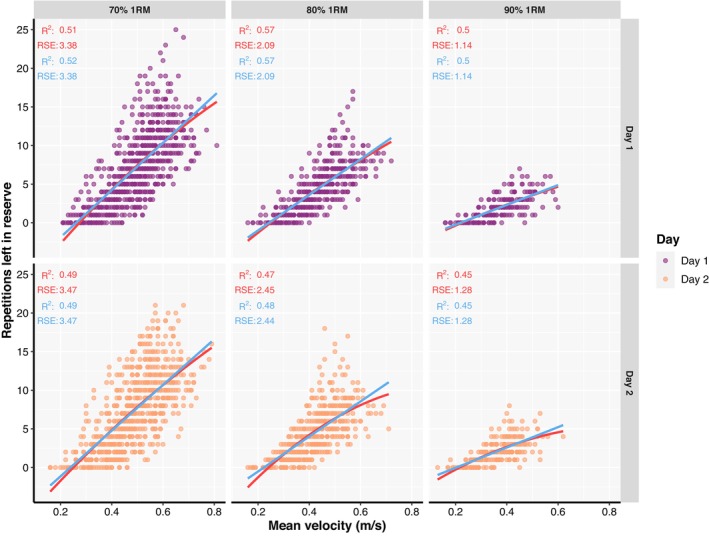
A general relationship between repetitions left in reserve and mean absolute velocity fitted with linear (red line) and second order polynomial regression (light blue line) models. Coefficient of determination (*R* ^2^), as well as residual standard errors (RSE), are also presented for both linear (in red) and second order polynomial regression (in light blue) models.

**FIGURE 2:**
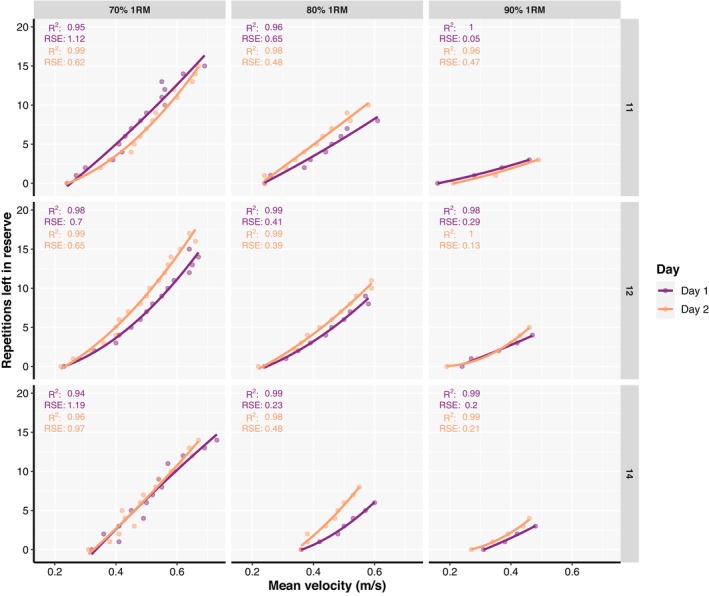
Individual relationships between repetitions left in reserve and mean absolute velocity fitted with second order polynomial regression models for representative participants. Coefficient of determination (*R* ^2^) as well as residual standard errors (RSE) are also presented for both testing sessions.

The goodness of fit for the general RIR‐velocity relationship across loads and testing sessions was generally strong and almost identical for both linear and second order polynomial regression models (Figure 1). However, the goodness of fit of the individual relationships was always stronger (Figure 2), regardless of the load, testing session, and regression model.

The goodness of fit of individual RIR‐velocity relationships was affected by testing session, model type, and training experience (Table 1). *R* ^*2*^ was higher in the second testing session (*p* < 0.001), when the second order polynomial regression model was used (*p* < 0.001), and with 90% (*p* = 0.030) and 80% (*p* = 0.030) compared to 70% of 1RM load (Appendix S3). RSE was higher in the second testing session (*p* < 0.001), as the load decreased from 90% to 70% 1RM (*p* < 0.001) and among less experienced individuals (*p* = 0.035).

**TABLE 1:** Factors affecting the goodness of fit of individual RIR‐velocity relationships.

Predictors	*R* ^2^			RSE		
	Estimates	CI	*p*	Estimates	CI	*p*
(Intercept)	0.97	0.77 to 1.16	<0.001	0.91	−0.02 to 1.85	0.056
Day [Day 2]	−0.02	−0.04 to −0.01	0.007	0.11	0.05 to 0.17	<0.001
Polynomial model	0.03	0.02 to 0.05	<0.001	−0.06	−0.12 to 0.00	0.056
Sex [male]	0.04	−0.02 to 0.10	0.234	−0.26	−0.55 to 0.03	0.076
Load [80% 1RM]	0.02	0.00 to 0.04	0.017	−0.58	−0.65 to −0.51	<0.001
Load [90% 1RM]	0.03	0.01 to 0.04	0.009	−0.86	−0.93 to −0.78	<0.001
Emotional Stability	−0.00	−0.00 to 0.00	0.432	0.01	−0.00 to 0.03	0.130
Conscientiousness	−0.00	−0.01 to 0.00	0.315	0.00	−0.01 to 0.02	0.730
Training experience [>3 years]	0.04	−0.00 to 0.09	0.071	−0.24	−0.46 to −0.02	0.035
Habitual loads [70–80% 1RM]	0.02	−0.04 to 0.07	0.613	−0.07	−0.36 to 0.21	0.627
Habitual loads [>80% 1RM]	0.04	−0.03 to 0.11	0.234	−0.07	−0.39 to 0.25	0.679
Habitual repetitions [8–12]	0.02	−0.03 to 0.08	0.403	−0.05	−0.30 to 0.20	0.691
Habitual repetitions [>12]	−0.00	−0.06 to 0.06	0.987	0.14	−0.14 to 0.42	0.328
Habitual repetitions in reserve [>2 RIR]	0.02	−0.03 to 0.06	0.490	0.04	−0.19 to 0.26	0.728
Relative strength (1RM/BM)	−0.04	−0.10 to 0.03	0.253	0.17	−0.14 to 0.49	0.284
Random Effects						
σ^2^	0.01			0.12		
τ_00 ID_	0.00			0.09		
ICC	0.30			0.42		
N _ID_	46			46		
Observations	552			524		
Marginal *R* ^2^/Conditional *R* ^2^	0.139/0.393			0.458/0.686		

*Note*: Reference groups were the following: Load [70% 1RM], sex [female], training experience [<3 years], habitual loads [< 70% 1RM], habitual repetitions [<8 repetitions], and habitual repetitions in reserve [<2 RIR]. Number of observations for the RSE model is lower than for the *R* ^2^ model as some polynomial model with 90% of 1RM resulted in no error (i.e., perfect fit due to the minimum number of data points, ~ 3). These observations were automatically discarded by the model.

Abbreviations: 1RM, one repetition maximum; BM, body mass; CI, 95% confidence intervals; ICC, intraclass correlation coefficient; *p*, *p* value; R^2^, coefficient of determination; RIR, repetitions in reserve; RSE, residual standard error.

**FIGURE 3:**
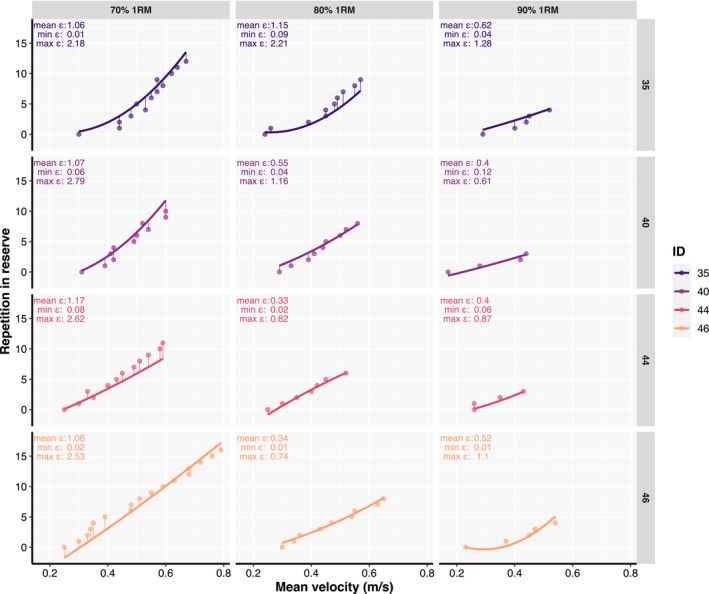
Individual relationships between repetitions left in reserve and mean absolute velocity established with the second order polynomial regression in the first testing session (line) and fitted to the data of the second testing session (dots). Thin vertical lines connecting thick lines and dots across participants represent distances (or residual errors) between predicted and observed data. Mean (mean ε), minimal (min ε), and maximal (max ε) errors are also presented for each participant and load. This data is from representative participants where models' prediction errors never exceeded a two repetitions error mark.

Predictive validity of the general RIR‐velocity relationship was acceptable for 80% and 90% but not for 70% of 1RM (Appendix S4). Regardless of the load used and whether linear or polynomial models were fitted to the data, the predictive validity of individual RIR‐velocity relationships was always acceptable since the absolute error between observed and predicted data on the second testing session—while using the data of the first testing session to make predictions—was always lower than two repetitions (Figure 3; Appendix S3). The linear mixed‐effects model investigating factors affecting absolute differences between observed and predicted data on the second testing session revealed only load was an influential factor (Table 2), with absolute errors decreasing as the load increased (*p* < 0.001). Similarly, the generalized linear mixed‐effects model examining factors affecting the probability of not exceeding an absolute prediction error of two repetitions revealed load, sex, and number of repetitions typically performed per set were influential factors (Table 2). Specifically, the probability of exceeding the two repetitions prediction error linearly decreased as the load increased (*p* < 0.001), was greater for females than males (*p* = 0.003), and greater among participants who usually completed more than 12 repetitions compared to those who usually performed less than eight repetitions during their own training (*p* = 0.04; Appendix S3).

**TABLE 2:** Factors affecting the (1) the absolute differences between predicted and observed RIR in a subsequent testing session based on individual RIR‐velocity relationships; and (2) the probability of individual RIR‐velocity relationships exceeding a prediction error of two repetitions.

Predictors	Absolute differences			Exceeding the error		
	Estimates	CI	*p*	OR	CI	*p*
(Intercept)	1.10	−0.32 to 2.53	0.130	0.02	0.00–0.25	0.002
Sex [male]	−0.29	−0.74 to 0.17	0.215	0.30	0.14–0.67	0.003
Polynomial model	−0.03	−0.13 to 0.07	0.580	0.97	0.80–1.16	0.714
Load [80% 1RM]	−0.41	−0.52 to 0.30	<0.001	0.63	0.51–0.77	<0.001
Load [90% 1RM]	−0.81	−0.95 to −0.67	<0.001	0.24	0.17–0.33	<0.001
Emotional stability	0.01	−0.02 to 0.04	0.697	1.03	0.99–1.08	0.151
Conscientiousness	0.01	−0.02 to 0.05	0.462	1.04	0.99–1.09	0.095
Training experience [>3 years]	−0.11	−0.55 to 0.34	0.633	0.78	0.42–1.43	0.424
Habitual repetitions [8–12]	0.00	−0.51 to 0.51	0.992	1.27	0.63–2.55	0.508
Habitual repetitions [>12]	0.56	−0.00 to 1.13	0.051	2.65	1.23–5.74	0.013
Habitual loads [70%–80% 1RM]	0.26	−0.31 to 0.82	0.371	0.90	0.42–1.95	0.787
Habitual loads [>80% 1RM]	0.12	−0.54 to 0.77	0.723	1.16	0.48–2.79	0.747
Habitual repetitions in reserve [>2 RIR]	0.14	−0.32 to 0.59	0.552	1.18	0.64–2.18	0.593
Random effects						
σ^2^	1.70			3.29		
τ_00 ID_	0.37			0.61		
ICC	0.18			0.16		
N _ID_	46			46		
Observations	2607			2607		
Marginal *R* ^2^/Conditional *R* ^2^	0.099/0.261			0.147/0.281		

*Note*: Reference groups were the following: Load [70% 1RM], sex [female], training experience [<3 years], habitual loads [<70% 1RM], habitual repetitions [<8 repetitions], and habitual repetitions in reserve [<2 RIR].

Abbreviations: 1RM, one repetition maximum; BM, body mass; CI, 95% confidence intervals; OR, odds ratio; *p*, *p* value; R^2^, coefficient of determination; RIR, repetitions in reserve; RIR‐velocity relationship, relationships between repetitions in reserve and their mean velocity; RSE, residual standard error.

**FIGURE 4:**
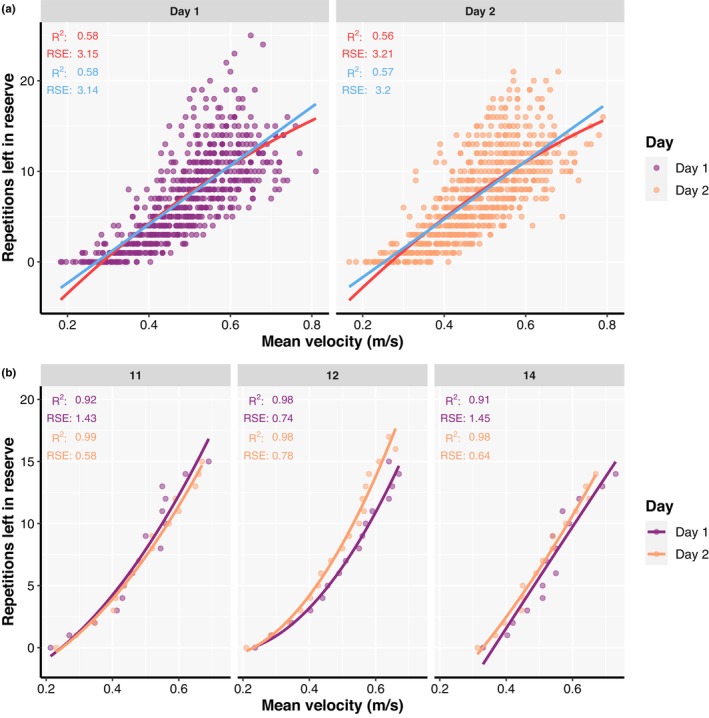
General relationship (a) between repetitions left in reserve and mean absolute velocity fitted with linear (red line) and second order polynomial regression (light blue line) models. Individual relationships (b) between repetitions left in reserve and mean absolute velocity fitted with second order polynomial regression models for representative participants. Velocities associated with different repetitions left in reserve were averaged across 70%, 80%, and 90% of 1RM for both general (a) and individual (b) relationships in this figure. Coefficient of determination (*R* ^2^), as well as residual standard errors (RSE), are also presented for both testing sessions and relationships.

When the overlapping RIRs across sets performed to failure with different loads were averaged, general and individual RIR‐velocity relationships' goodness of fit was generally comparable to relationships fitted for each load separately (Figure 4; Appendix S5). Similarly, the goodness of fit of individual RIR‐velocity relationships was affected by the same factors as previously reported—apart from training experience which was not a significant predictor in these models—for relationships established for each load separately (Appendix S5). The predictive validity of individual but not general RIR‐velocity relationships averaged across loads was acceptable, with comparable prediction errors for both linear and polynomial models (Appendix S5). Finally, models investigating factors affecting the predictive validity of individual RIR‐velocity relationships averaged across loads revealed only habitual repetitions practices were influential factors, similar to the results reported for RIR‐velocity relationships established for each load separately (Appendix S5).

## 4 DISCUSSION

To our knowledge, this is the first study that examined the goodness of fit and prediction accuracy of general and individual RIR‐velocity relationships in a free‐weight back squat exercise while also exploring the effects of modeling strategies, sex, training status and history, as well as personality traits on goodness of fit and accuracy of those relationships. The main findings of this study were (1) individual rather than general RIR‐velocity relationships yielded a higher goodness of fit for all loads and on both testing sessions; (2) goodness of fit for both individual and general relationships was higher among more experienced compared to less experienced individuals and with higher (i.e., 90% and 80% 1RM) compared to lower loads (i.e., 70% 1RM), but was not affected by sex, training practices nor personality traits; (3) for individual RIR‐velocity relationships, second order polynomial regression models yielded a better goodness of fit compared to linear models whereas both regression models fit the data equally well for the general RIR‐velocity relationship; (4) individual, but not general, RIR‐velocity relationships displayed acceptable *RSE*, regardless of the load or testing session; (5) individual RIR‐velocity relationships established in the first testing session always provided acceptable predictions of RIR in the second testing session, regardless of the load, whereas general RIR‐velocity relationships yielded acceptable prediction errors only with 80% and 90% 1RM loads; (6) prediction accuracy was only affected by sex and habitual repetition practices in models from males, and those performing less than 12 repetitions during their own training, having a slightly lower prediction accuracy; and (7) goodness of fit and prediction accuracy of individual RIR‐velocity relationships averaged across loads were comparable to the ones observed for the relationships established for each load separately. Therefore, these findings support the use of individual RIR‐velocity relationships for free‐weight RT monitoring and prescription, potentially improving adaptation and fatigue management.

Instead of prescribing a given, fixed number of repetitions per set, some authors recommended that each training set should be terminated when a given VL is reached due to a strong relationship that exists between VL and the percentage of performed repetitions out of the maximum possible for different Smith machine exercises (González‐Badillo et al., 2017). However, more recently researchers reported methodological issues with this concept with implications for RT volume prescription (García‐Ramos et al., 2021). For instance, even if a percentage of completed repetitions out of the maximum possible can be determined based on VL experienced in a set, the exact number of RIR remains unknown. This is important since the last repetitions of a set contribute more to the alteration of muscle energy balance and the abrupt increase in metabolites (Gorostiaga et al., 2012, 2014; Sánchez‐Medina & González‐Badillo, 2011). A very practical approach to monitoring RIR is through the use of the RIR‐based RPE scale (Helms et al., 2016) whereby individuals estimate how many additional repetitions they could have performed after finishing a training set. However, this method is subjective, and its accuracy can depend upon prior RT experience and is less accurate when training farther from failure and with moderate‐to‐high repetition sets (Ormsbee et al., 2019; Zourdos et al., 2021). To address this problem, García‐Ramos et al. (2018) formally established the general RIR‐velocity relationship in a Smith machine bench press exercise whereas Morán‐Navarro et al. (2019) examined velocities associated with 2, 4, 6, and 8 RIR in several Smith machine exercises. Both research groups observed some variability—expressed with coefficients of variation—in velocities associated with different RIR which was especially noticeable for upper body exercises (García‐Ramos et al., 2018; Morán‐Navarro et al., 2019) and less experienced individuals (Morán‐Navarro et al., 2019). In agreement with their observations, the goodness of fit for individual RIR‐velocity relationships was considerably greater in the present study, with *R* ^*2*^ values being twice as high for individual compared to general RIR‐velocity relationships in the free‐weight back squat. The current study also aimed to examine factors affecting the goodness of fit of individual RIR‐velocity relationships. Only the load and training experience affected the goodness of fit, with less experienced individuals' RIR‐velocity relationships having a lower *RSE* (*p* = 0.035; Table 1) compared to more experienced people. In addition, RIR‐velocity relationships with greater loads generally displayed lower *RSE* (*p* < 0.001; Table 1). However, it should be noted that the median *RSE* for less experienced individuals (*RSE* ≤1.67) and RIR‐velocity relationships with lower loads (*RSE* ≤1.18) was still generally low (even lower when fitted with polynomial regression models) and comparable to more experienced people which was also mirrored by similar *R* ^*2*^ values for the models fit. Considering this, and the lack of significant effects from training status, history, and personality traits on the stability of individual RIR‐velocity relationships, these findings could likely be generalized to the resistance‐trained population using free‐weight exercises.

To gauge the practical usefulness of RIR‐velocity relationships, their predictive validity must be evaluated. Thus, the present study investigated the ability of general and individual RIR‐velocity relationships established in an initial testing session to predict RIR in a subsequent testing session. Despite comparatively weak goodness of fit of general RIR‐velocity relationships, their predictive validity was unacceptable only for 70% of 1RM (>2 repetitions). In contrast, regardless of the load and modeling strategy used, individual RIR‐velocity relationships displayed lower, acceptable mean prediction errors. These prediction errors of individual RIR‐velocity relationships were affected by sex, load, and habitual repetitions practices (Table 2). While examination of prediction errors by sex and load in isolation always yielded mean prediction errors lower than two repetitions, females tended to have slightly higher prediction errors than the predetermined criteria with lower loads. Perhaps, the fact that females had somewhat lower relative strength than males in this study could partially explain slightly higher prediction errors. Additionally, individuals who typically performed more than 12 repetitions during their own training had higher than two repetitions prediction errors with 70% of 1RM. This suggests that the accuracy of individual RIR‐velocity relationships might be slightly compromised with lower loads (e.g., 70% 1RM), especially with individuals who usually perform many repetitions during their training sets. It may be that those individuals generally possess a greater variability in performance for reasons unexplored in the present study. Finally, it should be noted that polynomial regression models should be used to maximize prediction accuracy. Nevertheless, individual, but not general RIR‐velocity relationships always demonstrated high goodness of fit and acceptable accuracy and thus can be used for RT monitoring and prescription.

Despite acceptable mean prediction accuracy being observed for individual RIR‐velocity relationships in the present study, it is important to note that models' prediction errors might not be acceptable for all individuals, or RIRs thereof. For instance, Figure 3 illustrates four individuals whose models' prediction errors were on average almost perfect (~ 1 repetition). However, a maximal prediction error for individual RIR should also be appreciated from the figure. This is important as only focusing on the mean error across different RIRs can hide the inability of the model to accurately predict the entire RIR‐velocity spectrum. Furthermore, a thorough inspection of the descriptive data of average prediction errors revealed that females, less strong individuals, and those with less than 3 years of RT experience, tended to have slightly higher mean prediction errors compared to their respective counterparts (Appendix S4). Therefore, individual RIR‐velocity relationships should be evaluated, and their usefulness determined on a case‐by‐case basis, with special attention given to less experienced and weaker individuals.

The present study also attempted to evaluate both general and individual RIR‐velocity relationships averaged across the loads. For this purpose, velocities of overlapping RIR (i.e., different RIR not unique across the three loads used in this study) were averaged to examine whether such RIR‐velocity relationship could yield high goodness of fit and acceptable prediction accuracy. Indeed, with velocities associated with overlapping RIRs averaged across loads both general and individual RIR‐velocity relationships' goodness of fit (Figure 4) was generally comparable to relationships fitted for each load separately (Figure 1; Figure 2). Similarly, the predictive validity of individual but not general RIR‐velocity relationships averaged across loads was acceptable, with comparable prediction errors for both linear and polynomial models (Appendix S5). Finally, the results of the models investigating factors affecting both goodness of fit and predictive validity of individual RIR‐velocity relationships averaged across loads were similar to results reported for RIR‐velocity relationships established for each load separately (Appendix S5). Therefore, the process of establishing and using RIR‐velocity relationships in practice could further be simplified by establishing a single relationship which covers a range of loads (i.e., 70%–90% of 1RM), rather than having a profile for each load separately. This opens another possibility of using individual RIR‐velocity relationships which goes beyond set termination and equating for effort across individuals when prescribing RT. For example, a training program could call for three sets of six repetitions at 2RIR rather than assigning a percentage (e.g., 85% of 1RM) to the prescribed repetitions. Then, the load could be selected based on the “starting velocity” (i.e., the velocity associated with 6RIR) and the set terminated when the velocity associated with 2RIR is reached (i.e., a “stopping velocity”). Furthermore, this approach allows for autoregulation of training loads which can be adjusted for subsequent sets (or workouts) to meet the training goal; this is important when considering possible day‐to‐day variations in performance. Essentially, the proposed ways in which RIR‐based RPE can be used to autoregulate load selection and set termination (Helms et al., 2016; Ormsbee et al., 2019) can also be performed using individual RIR‐velocity relationships, but with greater accuracy, especially among individuals who may be worse at subjectively gauging RIR. Therefore, the individual RIR‐velocity relationship can be used in lieu of traditional RT methods to monitor, prescribe, and adjust both the training load and set‐volume more accurately.

The present study comprehensively examined the goodness of fit and predictive validity of RIR‐velocity relationships for monitoring and prescribing RT while also exploring the effects of a range of factors on these relationships. However, there are several considerations worth noting when interpreting the results. First, it is unknown whether the findings of the present study extend beyond free weight squats and transfer to other exercises. However, even if other exercises prove less or more reliable, it is likely that the effects of training history and status, sex, and personality are similar across exercises. Second, all participants in the present study had at least 6 months of RT experience, so the extent to which the current findings translate to those without RT experience is unknown. Third, the number of females included in the analysis was considerably lower than males, so the male data may be more generalizable. With that said, the females in the present study had a wide range of strength levels, training experience, and habitual training practices, possibly improving the sample's generalizability. Finally, it is important to highlight that a different choice for the acceptable repetition error threshold for the RIR‐velocity relationships could have influenced the findings and subsequent interpretation. However, a RT monitoring and prescription method with higher than a two repetitions error, especially one that has a financial cost, may not be justified in practical settings, given this would be higher than the average error of subjectively rated RIR in most cases (Halperin et al., 2021).

## 5 CONCLUSION

The present study is the first to thoroughly examine the utility of general and individual RIR‐velocity relationships with free‐weight exercises. Regardless of the load and modeling strategy used, individualized RIR‐velocity relationships provided twice as high goodness of fit and were always able to predict RIR in a subsequent testing session with acceptable accuracy compared to general RIR‐velocity relationships. Females, less strong individuals, and those with less than 3 years of RT experience tended to have slightly higher mean prediction errors compared to their respective counterparts. However, the prediction error for females was only slightly higher with respect to the predetermined criteria, and only present with lower loads. Therefore, individual RIR‐velocity relationships should be evaluated, and their usefulness determined on a case‐by‐case basis, with special attention given to less experienced and weaker individuals. Using RIR‐velocity relationships in practice could further be simplified by establishing a single relationship which covers a range of loads (i.e., 70% to 90% of 1RM), rather than having a profile for each load separately. This allows selection of the loads based on the “starting velocity” (i.e., the velocity associated with an indented load that can be lifted a given number of times) and termination of the sets when the “stopping velocity” (i.e., velocity associated with an intended RIR) is reached. Therefore, the individual RIR‐velocity relationship can be used in lieu of traditional RT methods to monitor, prescribe, and adjust both the training load and set‐volume more accurately, potentially allowing for more efficient adaptation and better fatigue management.

## AUTHOR CONTRIBUTIONS

IJ, MRM, and ERH designed the study. IJ and KP conducted the study. IJ performed all the analyses, visualized the data, and wrote the first draft of the manuscript. All authors edited and revised the manuscript and approved the final version of the manuscript.

## FUNDING INFORMATION

I.J. was supported by the AUT Vice Chancellor's Doctoral Scholarship. No other sources of funding were used in the preparation of this manuscript. Open Access funding enabled and organized by CAUL and its Member Institutions.

## ETHICS STATEMENT

The protocol of this study was approved by and was in accordance with the ethical requirements of the Auckland University of Technology Ethics Committee (approval number: 20/55). All methods were conducted in strict adherence to the Code of Ethics of the World Medical Association (Declaration of Helsinki).

## Supporting information


Appendix S1.

Appendix S2.

Appendix S3.

Appendix S4.

Appendix S5.

## Data Availability

The dataset and analysis code scripts are available at the Open Science Framework (URL: https://osf.io/5ejcp/).
