# Supplementary material for: Modeling the repetitions‐in‐reserve‐velocity relationship: a valid method for resistance training monitoring and prescription, and fatigue management
Source: Physiol Rep. 2024 Feb 28;12(5):e15955. doi: 10.14814/phy2.15955 (PMC10901726; doi:10.14814/phy2.15955)
Supplement: Supplementary file 2 — Appendix S2. [file PHY2-12-e15955-s001.docx]

Jukic et al. (2023). Modelling the repetitions-in-reserve-velocity relationship is a valid method for resistance training monitoring and prescription and fatigue control. *Physiological Reports*. Email the corresponding author: ivan.jukic@aut.ac.nz. Sport Performance Research Institute New Zealand (SPRINZ), Auckland University of Technology, Auckland, New Zealand

**Supplementary file II: Details on model specification and diagnostics**

A second order polynomial regression always yielded a slightly better goodness of fit compared to simple liner regression for modelling the individual relationship between repetitions left in reserve (RIR) and their mean velocity (i.e., RIR-velocity relationship). Therefore, polynomial regression-based models were used for all figures in the manuscript. However, since one of the aims was to see whether less complex linear models can yield acceptable predictive validity of RIR, predictive validity of both linear and polynomial regression models was subsequently evaluated. Importantly, while evaluating absolute differences between observed and predicted RIRs several individual predictions (5 out of 2612 observations) yielded unrealistic values. Therefore, these outliers were removed before examining the influential factors on absolute differences between predicted and observed RIRs and the likelihood of exceeding prediction errors higher than 2 repetitions as the inclusion of outliers affected the stability of the models.

Since regression-based models can be sensitive to variables that are correlated, the variance inflation factors for all predictor parameters used in the linear mixed-effects model were inspected to check for multi-collinearity. For linear mixed-effects models, a Gaussian distribution was assumed, and the approximate normal distribution of model residuals was checked to confirm goodness of fit. To ensure the assumptions of the model were met, the plotted residuals were also checked to ensure homoscedasticity prior to utilising the results of the model. Model investigating the factors potentially affecting the *R^2^* values of individual RIR-velocity relationships had a slightly skewed distribution of residuals (i.e., the extreme sample quantiles deviated from the diagonal line in the Q-Q plot). However, this model was retained for two reasons. Firstly, linear mixed effects models were shown to be robust to violations of distributional assumptions. Secondly, when mixed-effects models with beta distribution were fitted to inspect the robustness of this model’s estimates, the conclusions remained largely the same (i.e., only the p value for training experience predictor became lower). To validate the assumptions of the generalised mixed-effects model, a simulation-based approach based on examination of standardised residuals was used to test for uniformity of residuals, under and over dispersion, and outliers. None of the tests revealed issues with the models fit.
