# Supplementary material for: Modeling the repetitions‐in‐reserve‐velocity relationship: a valid method for resistance training monitoring and prescription, and fatigue management
Source: Physiol Rep. 2024 Feb 28;12(5):e15955. doi: 10.14814/phy2.15955 (PMC10901726; doi:10.14814/phy2.15955)
Supplement: Supplementary file 3 — Appendix S3. [file PHY2-12-e15955-s005.docx]

Jukic et al. (2023). Modelling the repetitions-in-reserve-velocity relationship is a valid method for resistance training monitoring and prescription and fatigue control. *Physiological Reports*. Email the corresponding author: ivan.jukic@aut.ac.nz. Sport Performance Research Institute New Zealand (SPRINZ), Auckland University of Technology, Auckland, New Zealand

**Supplementary file III: Pairwise comparisons for significant, categorical variables with more than 2 levels for all outcomes of interest.**

Table 1. Pairwise comparisons with Holm-Bonferroni correction for the effects of loads on the coefficient of determination for the individual relationships between repetitions in reserve and their mean velocity

| *contrast* | *estimate* | *SE* | *t.value* | *p.value* |
| --- | --- | --- | --- | --- |
| 70% / 80% | -0.02 | 0.01 | -2.39 | 0.03 |
| 70% / 90% | -0.03 | 0.01 | -2.62 | 0.03 |
| 80% / 90% | 0.00 | 0.01 | -0.22 | 0.82 |

Table 2. Pairwise comparisons with Holm-Bonferroni correction for the effects of loads on the residual standard error for the individual relationships between repetitions in reserve and their mean velocity

| *contrast* | *estimate* | *SE* | *t.value* | *p.value* |
| --- | --- | --- | --- | --- |
| 70% / 80% | 0.58 | 0.04 | 16.09 | < 0.001 |
| 70% / 90% | 0.86 | 0.04 | 22.54 | < 0.001 |
| 80% / 90% | 0.28 | 0.04 | 7.24 | < 0.001 |

Table 3. Pairwise comparisons with Holm-Bonferroni correction for the effects of loads on the predictive validity (i.e., absolute errors) of individual relationships between repetitions in reserve and their mean velocity

| *contrast* | *estimate* | *SE* | *t.value* | *p.value* |
| --- | --- | --- | --- | --- |
| 70% / 80% | 0.41 | 0.06 | 7.14 | < 0.001 |
| 70% / 90% | 0.81 | 0.07 | 11.03 | < 0.001 |
| 80% / 90% | 0.40 | 0.08 | 5.12 | < 0.001 |

Table 4. Pairwise comparisons with Holm-Bonferroni correction for the effects of training practices related to the loads used on the predictive validity (i.e., absolute errors) of individual relationships between velocity loss and the percentage of the completed repetitions with respect to the maximum possible

| *contrast* | *estimate* | *SE* | *t.value* | *p.value* |
| --- | --- | --- | --- | --- |
| 70% / 70 – 80% | 3.09 | 2.04 | 1.51 | 0.28 |
| < 70% / > 80% | 5.48 | 2.32 | 2.36 | 0.07 |
| 70 – 80% / > 80% | 2.39 | 1.72 | 1.39 | 0.28 |

Table 5. Pairwise comparisons with Holm-Bonferroni correction for the effects of loads on the probability of individual repetitions in reserve-velocity relationships not exceeding a prediction error of 2 repetitions

| *contrast* | *odds.ratio* | *SE* | *z.value* | *p.value* |
| --- | --- | --- | --- | --- |
| 70% / 80% | 1.59 | 0.17 | 4.43 | < 0.001 |
| 70% / 90% | 4.20 | 0.68 | 8.91 | < 0.001 |
| 80% / 90% | 2.65 | 0.45 | 5.76 | < 0.001 |

Table 6. Pairwise comparisons with Holm-Bonferroni correction for the effects of training practices related to the number of repetitions participants do during their own training on the probability of individual repetitions in reserve-velocity relationships not exceeding a prediction error of 2 repetitions.

| *contrast* | *odds.ratio* | *estimate* | *SE* | *z.value* | *p.value* |
| --- | --- | --- | --- | --- | --- |
| < 8 repetitions / 8 – 12 repetitions | 0.79 | 0.28 | 0.36 | -0.66 | 0.51 |
| < 8 repetitions / > 8 – 12 repetitions | 0.38 | 0.15 | 0.65 | -2.48 | 0.04 |
| 8 – 12 repetitions / > 12 repetitions | 0.48 | 0.17 | 0.39 | -2.10 | 0.07 |
