# Supplementary material for: Modeling the repetitions‐in‐reserve‐velocity relationship: a valid method for resistance training monitoring and prescription, and fatigue management
Source: Physiol Rep. 2024 Feb 28;12(5):e15955. doi: 10.14814/phy2.15955 (PMC10901726; doi:10.14814/phy2.15955)
Supplement: Supplementary file 4 — Appendix S4. [file PHY2-12-e15955-s003.docx]

Jukic et al. (2023). Modelling the repetitions-in-reserve-velocity relationship is a valid method for resistance training monitoring and prescription and fatigue control. *Physiological Reports*. Email the corresponding author: ivan.jukic@aut.ac.nz. Sport Performance Research Institute New Zealand (SPRINZ), Auckland University of Technology, Auckland, New Zealand

**Supplementary file IV: Absolute differences between predicted and observed repetitions in reserve in the second testing session using the models established in the first testing session.**

Table 1. Prediction errors of general and individual repetitions in reserve-velocity relationships fitted with linear and polynomial regression models

|  | *Linear model* | | | | | | | | *Polynomial model* | | | | | | | |
| --- | --- | --- | --- | --- | --- | --- | --- | --- | --- | --- | --- | --- | --- | --- | --- | --- |
|  | *general* | | | | *individual* | | | | *general* | | | | *individual* | | | |
| *Load* | *ε* | *Min ε* | *Max ε* | *ε* | | *Min ε* | *Max ε* | *ε* | | *Min ε* | *Max ε* | *ε* | | *Min ε* | *Max ε* |  |
| 70% 1RM | 2.67 | 0.01 | 11.56 | 1.94 | | 0.00 | 8.66 | 2.68 | | 0.02 | 11.50 | 1.89 | | 0.01 | 8.90 |  |
| 80% 1RM | 1.83 | 0.00 | 13.01 | 1.55 | | 0.00 | 7.86 | 1.83 | | 0.00 | 12.94 | 1.48 | | 0.00 | 7.76 |  |
| 90% 1RM | 0.97 | 0.00 | 5.56 | 1.07 | | 0.00 | 4.01 | 0.96 | | 0.01 | 5.53 | 1.16 | | 0.00 | 6.00 |  |

Note. 1RM, one-repetition maximum; Mean ε, mean error; Min ε, minimal error; Max ε, maximal error.

Table 2. Prediction errors of general and individual repetitions in reserve-velocity relationships fitted with linear regression models broken down by sex, load, and training experience

|  |  | *females* | | | | | | | *males* | | | | | |
| --- | --- | --- | --- | --- | --- | --- | --- | --- | --- | --- | --- | --- | --- | --- |
|  |  | *general* | | | | *individual* | | | *general* | | | *individual* | | |
| *Load* | *Experience* | | *ε* | *Min ε* | *Max ε* | *ε* | *Min ε* | *Max ε* | *ε* | *Min ε* | *Max ε* | *ε* | *Min ε* | *Max ε* |
| 70% 1RM | <3 | | 3.17 | 0.05 | 11.56 | 2.01 | 0.03 | 5.79 | 2.75 | 0.01 | 8.19 | 2.16 | 0.01 | 8.66 |
|  | >3 | | 3.44 | 0.01 | 9.48 | 2.07 | 0.00 | 7.81 | 1.95 | 0.01 | 7.52 | 1.66 | 0.00 | 8.64 |
| 80% 1RM | <3 | | 2.75 | 0.00 | 13.01 | 2.12 | 0.00 | 7.86 | 1.84 | 0.00 | 7.39 | 1.43 | 0.03 | 7.81 |
|  | >3 | | 2.03 | 0.07 | 5.55 | 1.99 | 0.07 | 4.96 | 1.24 | 0.07 | 4.24 | 1.17 | 0.03 | 4.02 |
| 90% 1RM | <3 | | 1.27 | 0.03 | 5.56 | 1.53 | 0.01 | 3.87 | 1.03 | 0.00 | 3.43 | 1.13 | 0.01 | 4.01 |
|  | >3 | | 0.96 | 0.03 | 3.82 | 0.89 | 0.11 | 3.19 | 0.72 | 0.05 | 1.92 | 0.82 | 0.00 | 2.72 |

*Note:* ε – error; <3, less than 3 years; >3, more than 3 years

Table 3. Prediction errors of general and individual repetitions in reserve-velocity relationships fitted with linear regression models broken down by sex, load, and relative strength

|  |  | *females* | | | | | | | *males* | | | | | |
| --- | --- | --- | --- | --- | --- | --- | --- | --- | --- | --- | --- | --- | --- | --- |
|  |  | *general* | | | | *individual* | | | *general* | | | *individual* | | |
| *Load* | *Strength* | | *ε* | *Min ε* | *Max ε* | *ε* | *Min ε* | *Max ε* | *ε* | *Min ε* | *Max ε* | *ε* | *Min ε* | *Max ε* |
| 70% 1RM | Less strong | | 2.81 | 0.05 | 9.40 | 1.95 | 0.03 | 5.79 | 2.37 | 0.01 | 6.07 | 1.60 | 0.01 | 8.52 |
|  | Strong | | 3.67 | 0.01 | 11.56 | 2.11 | 0.00 | 7.81 | 2.30 | 0.01 | 8.19 | 1.97 | 0.00 | 8.66 |
| 80% 1RM | Less strong | | 1.91 | 0.00 | 6.62 | 2.27 | 0.16 | 5.83 | 1.69 | 0.07 | 4.07 | 1.40 | 0.03 | 3.83 |
|  | Strong | | 2.82 | 0.07 | 13.01 | 1.91 | 0.00 | 7.86 | 1.48 | 0.00 | 7.39 | 1.26 | 0.03 | 7.81 |
| 90% 1RM | Less strong | | 0.96 | 0.03 | 3.02 | 1.69 | 0.01 | 3.84 | 1.07 | 0.07 | 2.98 | 1.00 | 0.01 | 3.93 |
|  | Strong | | 1.24 | 0.03 | 5.56 | 0.94 | 0.02 | 3.87 | 0.83 | 0.00 | 3.43 | 0.97 | 0.00 | 4.01 |

*Note:* ε – error; Less strong, one-repetition maximum-body mass ratio lower than 1.25 and 1.50 for females and males, respectively; Strong, one-repetition maximum-body mass ratio greater than 1.25 and 1.50 for females and males, respectively.

Table 4. Prediction errors of general and individual repetitions in reserve-velocity relationships fitted with polynomial regression models broken down by sex, load, and training experience

|  |  | *females* | | | | | | | *males* | | | | | |
| --- | --- | --- | --- | --- | --- | --- | --- | --- | --- | --- | --- | --- | --- | --- |
|  |  | *general* | | | | *individual* | | | *general* | | | *individual* | | |
| *Load* | *Experience* | | *ε* | *Min ε* | *Max ε* | *ε* | *Min ε* | *Max ε* | *ε* | *Min ε* | *Max ε* | *ε* | *Min ε* | *Max ε* |
| 70% 1RM | <3 | | 3.16 | 0.04 | 11.50 | 1.83 | 0.05 | 6.62 | 2.74 | 0.02 | 7.98 | 2.12 | 0.03 | 8.48 |
|  | >3 | | 3.48 | 0.04 | 9.36 | 2.15 | 0.02 | 7.84 | 1.99 | 0.02 | 7.64 | 1.61 | 0.01 | 8.90 |
| 80% 1RM | <3 | | 2.76 | 0.00 | 12.94 | 2.05 | 0.02 | 7.76 | 1.85 | 0.00 | 7.39 | 1.38 | 0.00 | 7.67 |
|  | >3 | | 2.04 | 0.01 | 5.49 | 1.77 | 0.01 | 4.86 | 1.25 | 0.01 | 4.17 | 1.14 | 0.01 | 4.25 |
| 90% 1RM | <3 | | 1.21 | 0.04 | 5.53 | 1.65 | 0.04 | 5.96 | 1.04 | 0.03 | 3.40 | 1.18 | 0.00 | 6.00 |
|  | >3 | | 0.95 | 0.01 | 3.78 | 0.98 | 0.01 | 4.29 | 0.73 | 0.04 | 1.92 | 0.95 | 0.02 | 4.06 |

*Note:* ε – error; <3, less than 3 years; >3, more than 3 years

Table 5. Prediction errors of general and individual repetitions in reserve-velocity relationships fitted with polynomial regression models broken down by sex, load, and relative strength

|  |  | *females* | | | | | | | *males* | | | | | |
| --- | --- | --- | --- | --- | --- | --- | --- | --- | --- | --- | --- | --- | --- | --- |
|  |  | *general* | | | | *individual* | | | *general* | | | *individual* | | |
| *Load* | *Strength* | | *ε* | *Min ε* | *Max ε* | *ε* | *Min ε* | *Max ε* | *ε* | *Min ε* | *Max ε* | *ε* | *Min ε* | *Max ε* |
| 70% 1RM | Less strong | | 2.82 | 0.04 | 9.25 | 1.88 | 0.05 | 6.62 | 2.37 | 0.05 | 6.22 | 1.46 | 0.03 | 8.48 |
|  | Strong | | 3.69 | 0.04 | 11.50 | 2.07 | 0.02 | 7.84 | 2.31 | 0.02 | 7.98 | 1.95 | 0.01 | 8.90 |
| 80% 1RM | Less strong | | 1.94 | 0.00 | 6.61 | 2.20 | 0.04 | 6.23 | 1.71 | 0.01 | 4.14 | 1.37 | 0.03 | 4.43 |
|  | Strong | | 2.81 | 0.01 | 12.94 | 1.74 | 0.01 | 7.76 | 1.48 | 0.00 | 7.39 | 1.22 | 0.00 | 7.67 |
| 90% 1RM | Less strong | | 0.89 | 0.04 | 2.92 | 1.78 | 0.33 | 5.96 | 1.06 | 0.03 | 2.96 | 1.09 | 0.04 | 6.00 |
|  | Strong | | 1.22 | 0.01 | 5.53 | 1.06 | 0.01 | 4.36 | 0.84 | 0.04 | 3.40 | 1.06 | 0.00 | 6.00 |

*Note:* ε – error; Less strong, one-repetition maximum-body mass ratio lower than 1.25 and 1.50 for females and males, respectively; Strong, one-repetition maximum-body mass ratio greater than 1.25 and 1.50 for females and males, respectively.
