# Supplementary material for: Modeling the repetitions‐in‐reserve‐velocity relationship: a valid method for resistance training monitoring and prescription, and fatigue management
Source: Physiol Rep. 2024 Feb 28;12(5):e15955. doi: 10.14814/phy2.15955 (PMC10901726; doi:10.14814/phy2.15955)
Supplement: Supplementary file 5 — Appendix S5. [file PHY2-12-e15955-s002.docx]

Jukic et al. (2023). Modelling the repetitions-in-reserve-velocity relationship is a valid method for resistance training monitoring and prescription and fatigue control. *Physiological Reports*. Email the corresponding author: ivan.jukic@aut.ac.nz. Sport Performance Research Institute New Zealand (SPRINZ), Auckland University of Technology, Auckland, New Zealand

**Supplementary file V: Results of all the analyses related to the repetitions in reserve-velocity relationships where overlapping repetitions in reserve were averaged across the loads.**

Table 1. Goodness of fit for general and individual repetitions in reserve-velocity relationships fitted with linear and polynomial regression models

|  | | *general* | | | *individual* | | | | | |
| --- | --- | --- | --- | --- | --- | --- | --- | --- | --- | --- |
| *Day* | *Model type* | | *R^2^* | *RSE* | *Median R^2^* | *Min R^2^* | *Max R^2^* | *Median RSE* | *Min RSE* | *Max RSE* |
| 1 | linear | | 0.58 | 3.15 | 0.93 | 0.75 | 0.99 | 1.20 | 0.54 | 3.43 |
|  | polynomial | | 0.58 | 3.14 | 0.95 | 0.81 | 0.99 | 0.94 | 0.41 | 3.50 |
| 2 | linear | | 0.56 | 3.21 | 0.92 | 0.74 | 0.98 | 1.19 | 0.53 | 2.40 |
|  | polynomial | | 0.57 | 3.20 | 0.95 | 0.76 | 0.99 | 1.03 | 0.35 | 2.48 |

*Note:* R^2^, coefficient of determination; RSE, residual standard error

Table 2. Factors affecting the goodness of fit of individual repetitions in reserve-velocity relationships

|  | **R^2^** | | | **RSE** | | |
| --- | --- | --- | --- | --- | --- | --- |
| *Predictors* | *Estimates* | *CI* | *p* | *Estimates* | *CI* | *p* |
| (Intercept) | 0.91 | 0.79 – 1.04 | <0.001 | 0.95 | -0.14 – 2.04 | 0.087 |
| Day [Day 2] | -0.01 | -0.02 – 0.00 | 0.138 | 0.05 | -0.05 – 0.14 | 0.335 |
| Polynomial model | 0.02 | 0.01 – 0.03 | <0.001 | -0.14 | -0.24 – -0.05 | 0.004 |
| Sex [male] | 0.02 | -0.02 – 0.06 | 0.387 | -0.37 | -0.70 – -0.04 | 0.030 |
| Emotional Stability | -0.00 | -0.00 – 0.00 | 0.781 | 0.01 | -0.01 – 0.03 | 0.156 |
| Conscientiousness | -0.00 | -0.00 – 0.00 | 0.566 | -0.00 | -0.02 – 0.02 | 0.990 |
| Training experience  [> 3 years] | 0.02 | -0.01 – 0.06 | 0.112 | -0.15 | -0.41 – 0.11 | 0.257 |
| Habitual loads  [70 – 80% 1RM] | 0.00 | -0.04 – 0.04 | 0.884 | 0.05 | -0.28 – 0.38 | 0.772 |
| Habitual loads  [> 80% 1RM] | 0.01 | -0.03 – 0.05 | 0.626 | 0.23 | -0.14 – 0.61 | 0.218 |
| Habitual repetitions [8 – 12] | -0.00 | -0.04 – 0.03 | 0.801 | 0.15 | -0.15 – 0.44 | 0.321 |
| Habitual repetitions [> 12] | -0.02 | -0.06 – 0.02 | 0.298 | 0.44 | 0.12 – 0.77 | 0.008 |
| Habitual repetitions in reserve [> 2 RIR] | 0.01 | -0.02 – 0.04 | 0.670 | 0.20 | -0.06 – 0.46 | 0.139 |
| Relative strength (1RM/BM) | 0.01 | -0.03 – 0.05 | 0.699 | -0.10 | -0.47 – 0.27 | 0.581 |
| **Random Effects** | | | |  |  |  |
| σ^2^ | 0.00 | | | 0.11 | | |
| τ_00_ _ID_ | 0.00 | | | 0.10 | | |
| ICC | 0.41 | | | 0.49 | | |
| N _ID_ | 46 | | | 46 | | |
| Observations | 184 | | | 184 | | |
| Marginal R^2^ / Conditional R^2^ | 0.189 / 0.525 | | | 0.301 / 0.645 | | |

*Note:* Reference groups were the following: Load [70% 1RM], Sex [female], Training experience [< 3 years], Habitual loads [< 70% 1RM], Habitual repetitions [< 8 repetitions], Habitual repetitions in reserve [< 2 RIR]. 1RM, one repetition maximum; BM, body mass; RIR, repetitions in reserve; R^2^, coefficient of determination; RSE, residual standard error; ICC, intra-class correlation coefficient; CI, 95% confidence intervals; p, p value.

Table 3. Prediction errors of general and individual repetitions in reserve-velocity relationships fitted with linear and polynomial regression models

|  | *general* | | | *individual* | | |
| --- | --- | --- | --- | --- | --- | --- |
| *Model* | *ε* | *Min ε* | *Max ε* | *ε* | *Min ε* | *Max ε* |
| Linear | 2.45 | 0.01 | 11.34 | 1.62 | 0.00 | 8.30 |
| Polynomial | 2.46 | 0.00 | 11.25 | 1.56 | 0.00 | 8.25 |

*Note*: ε, mean error; min ε, minimum error; max ε, maximum error; RIR, repetitions in reserve.

Table 4. Factors affecting the 1) the absolute differences between predicted and observed RIR in a subsequent testing session based on individual RIR-velocity relationships; and 2) the probability of individual RIR-velocity relationships exceeding a prediction error of 2 repetitions.

|  | **Absolute differences** | | | **Exceeding the error** | | |
| --- | --- | --- | --- | --- | --- | --- |
| *Predictors* | *Estimates* | *CI* | *p* | *OR* | *CI* | *p* |
| (Intercept) | 0.54 | -1.59 – 2.66 | 0.621 | 0.07 | 0.00 – 2.10 | 0.126 |
| Sex [male] | -0.46 | -1.11 – 0.19 | 0.163 | 0.46 | 0.16 – 1.28 | 0.136 |
| Polynomial model | -0.06 | -0.19 – 0.08 | 0.393 | 0.93 | 0.71 – 1.21 | 0.573 |
| Emotional Stability | 0.01 | -0.03 – 0.04 | 0.746 | 1.01 | 0.95 – 1.07 | 0.708 |
| Conscientiousness | 0.01 | -0.02 – 0.05 | 0.454 | 1.04 | 0.98 – 1.10 | 0.227 |
| Training experience  [> 3 years] | -0.08 | -0.59 – 0.43 | 0.756 | 0.71 | 0.32 – 1.60 | 0.409 |
| Habitual repetitions [8 – 12] | -0.02 | -0.59 – 0.55 | 0.951 | 0.71 | 0.28 – 1.79 | 0.463 |
| Habitual repetitions [> 12] | 0.80 | 0.17 – 1.43 | 0.013 | 3.13 | 1.16 – 8.47 | 0.025 |
| Habitual loads  [70 – 80% 1RM] | 0.42 | -0.22 – 1.07 | 0.199 | 1.49 | 0.54 – 4.09 | 0.441 |
| Habitual loads  [> 80% 1RM] | 0.42 | -0.31 – 1.16 | 0.257 | 1.68 | 0.53 – 5.30 | 0.376 |
| Habitual repetitions in reserve  [> 2 RIR] | 0.23 | -0.28 – 0.74 | 0.379 | 1.52 | 0.66 – 3.49 | 0.328 |
| 1RM/BM | -0.02 | -0.74 – 0.70 | 0.959 | 0.74 | 0.23 – 2.35 | 0.609 |
| **Random Effects** | | | |  |  |  |
| σ^2^ | 1.56 | | | 3.29 | | |
| τ_00_ _ID_ | 0.45 | | | 0.98 | | |
| ICC | 0.22 | | | 0.23 | | |
| N _ID_ | 46 | | | 46 | | |
| Observations | 1346 | | | 1346 | | |
| Marginal R^2^ / Conditional R^2^ | 0.102 / 0.301 | | | 0.165 / 0.356 | | |

*Note*: Reference groups were the following: Load [70% 1RM], Sex [female], Training experience [< 3 years], Habitual loads [< 70% 1RM], Habitual repetitions [< 8 repetitions], Habitual repetitions in reserve [< 2 RIR]. 1RM, one repetition maximum; BM, body mass; RIR, repetitions in reserve; OR, odds ratio; R^2^, coefficient of determination; RSE, residual standard error; CI, 95% confidence intervals; p, p value; RIR-velocity relationship, relationships between repetitions in reserve and their mean velocity.

Table 5. Prediction errors of general and individual repetitions in reserve-velocity relationships fitted with linear regression models broken down by sex and training experience

|  |  | *general* | | | | *individual* | | |
| --- | --- | --- | --- | --- | --- | --- | --- | --- |
| *Sex* | *Experience* | | *ε* | *Min ε* | *Max ε* | *ε* | *Min ε* | *Max ε* |
| Female | <3 | | 2.98 | 0.02 | 11.34 | 1.84 | 0.01 | 6.29 |
|  | >3 | | 3.27 | 0.01 | 9.31 | 1.94 | 0.02 | 6.24 |
| Male | <3 | | 2.51 | 0.02 | 8.55 | 1.71 | 0.00 | 8.30 |
|  | >3 | | 1.71 | 0.01 | 7.23 | 1.26 | 0.01 | 7.38 |

*Note:* ε – error; <3, less than 3 years; >3, more than 3 years

Table 6. Prediction errors of general and individual repetitions in reserve-velocity relationships fitted with linear regression models broken down by sex and relative strength

|  |  | *general* | | | | *individual* | | |
| --- | --- | --- | --- | --- | --- | --- | --- | --- |
| *Sex* | *Strength* | | *ε* | *Min ε* | *Max ε* | *ε* | *Min ε* | *Max ε* |
| Female | Less strong | | 2.51 | 0.02 | 9.28 | 1.95 | 0.01 | 6.29 |
|  | Strong | | 3.57 | 0.01 | 11.34 | 1.84 | 0.02 | 6.24 |
| Male | Less strong | | 2.47 | 0.02 | 5.84 | 1.42 | 0.01 | 7.86 |
|  | Strong | | 1.95 | 0.01 | 8.55 | 1.48 | 0.00 | 8.30 |

*Note:* ε – error; Less strong, one-repetition maximum-body mass ratio lower than 1.25 and 1.50 for females and males, respectively; Strong, one-repetition maximum-body mass ratio greater than 1.25 and 1.50 for females and males, respectively.

Table 7. Prediction errors of general and individual repetitions in reserve-velocity relationships fitted with polynomial regression models broken down by sex and training experience.

|  |  | *general* | | | | *individual* | | |
| --- | --- | --- | --- | --- | --- | --- | --- | --- |
| *Sex* | *Experience* | | *ε* | *Min ε* | *Max ε* | *ε* | *Min ε* | *Max ε* |
| Female | <3 | | 2.94 | 0.03 | 11.25 | 1.74 | 0.05 | 6.20 |
|  | >3 | | 3.28 | 0.03 | 9.15 | 1.95 | 0.01 | 6.58 |
| Male | <3 | | 2.50 | 0.00 | 8.04 | 1.63 | 0.01 | 8.25 |
|  | >3 | | 1.77 | 0.00 | 7.03 | 1.21 | 0.00 | 7.10 |

*Note:* ε – error; <3, less than 3 years; >3, more than 3 years

Table 8. Prediction errors of general and individual repetitions in reserve-velocity relationships fitted with polynomial regression models broken down by sex and relative strength.

|  |  | *general* | | | | *individual* | | |
| --- | --- | --- | --- | --- | --- | --- | --- | --- |
| *Sex* | *Strength* | | *ε* | *Min ε* | *Max ε* | *ε* | *Min ε* | *Max ε* |
| Female | Less strong | | 2.50 | 0.03 | 9.08 | 1.84 | 0.05 | 6.20 |
|  | Strong | | 3.55 | 0.03 | 11.25 | 1.84 | 0.01 | 6.58 |
| Male | Less strong | | 2.47 | 0.00 | 5.33 | 1.27 | 0.01 | 7.94 |
|  | Strong | | 1.99 | 0.00 | 8.04 | 1.43 | 0.00 | 8.25 |

*Note:* ε – error; Less strong, one-repetition maximum-body mass ratio lower than 1.25 and 1.50 for females and males, respectively; Strong, one-repetition maximum-body mass ratio greater than 1.25 and 1.50 for females and males, respectively.
